# Cam Femoroacetabular Impingement as a Possible Explanation of Recalcitrant Anterior Knee Pain

**DOI:** 10.1155/2016/2064894

**Published:** 2016-05-10

**Authors:** Vicente Sanchis-Alfonso, Marc Tey, Joan Carles Monllau

**Affiliations:** ^1^Hospital 9 de Octubre and Hospital Arnau de Vilanova, Valencia, Spain; ^2^Hospital del Mar, Universitat Autònoma de Barcelona, Barcelona, Spain

## Abstract

We present a case of a patient with chronic anterior knee pain (AKP) recalcitrant to conservative treatment who returned to our office for severe hip pain secondary to Cam femoroacetabular impingement (Cam FAI) at 10 months after the onset of knee pain. This case highlights the fact that the main problem is not in the patella but in the hip in some patients with AKP. We hypothesize that there is an external femoral rotation in order to avoid the impingement and therefore the hip pain in patients with Cam FAI. This functional femoral rotation could provoke a patellofemoral imbalance that may be, in theory, responsible for patellofemoral pain in this particular patient. In our case, Cam FAI resolution was related to the resolution of AKP.

## 1. Introduction

We present a case of a patient with chronic anterior knee pain (AKP) recalcitrant to conservative treatment who returned to our office for severe hip pain secondary to Cam femoroacetabular impingement (Cam FAI) at 10 months after the onset of knee pain. In a previous paper, we found a link between Cam FAI and AKP in some young patients [1]. Our case highlights the fact that the main problem is not in the patella but in the hip in some patients with AKP [2–6]. We hypothesize that there is an external femoral rotation in order to avoid the impingement in patients with Cam FAI and therefore the hip pain. This functional femoral rotation could provoke a patellofemoral imbalance that might be, in theory, responsible for patellofemoral pain in this particular patient. The goal of this report is to analyze how Cam FAI resolution might be related to the resolution of AKP in our case.

## 2. Case Report

A 28-year-old female, who practiced athletics, came to our institution with a history of chronic severe anterior left knee pain. Pain onset was secondary to a direct traumatism of the knee from playing football one year earlier, increasing in intensity throughout that year until it became so intense that the patient visited our office for the first time (Visual Analogue Scale 8). She had great difficulties in driving her car because of the pain caused upon engaging the clutch, going downstairs, wearing high heels, and sitting with the bent knee for a long period of time (“movie sign”). The psychological evaluation that we routinely perform on our patients with AKP did not indicate anxiety, depression, kinesiophobia, or catastrophizing [7]. The physical therapy program (patellar taping, muscle training, and flexibility exercises) performed in our institution [7] was unsuccessful in improving her symptoms. This pain forced her to abandon the sports activities but she kept going to the gym. At this moment there was no pain in the left hip.

Ten months later, she came back to our office due to severe hip pain with no history of traumatism to justify it. The hip pain was so significant that it not only forced her to leave the gym but also made significant limitations in her regular daily activities. Moreover, she continued to suffer from knee pain. The Kujala Knee Score was 22 and the Nonarthritic Hip Score was 28.75. Preoperative pain intensity on the Visual Analogue Scale was 8 in both knee and hip. During physical examination of the hip there was a positive impingement test and a positive decompression test. A Dunn radiograph view showed an alpha angle of 58°. It is the angle between the line from the centre of the femoral head through the middle of the femoral neck and the line through the point where the contour of the femoral head-neck junction exceeds the radius of the femoral head. An angle >55° is considered indicative of Cam impingement [8]. The study by arthro-MRI of the left hip showed a Cam FAI and a detachment of the anterior labrum.

**Figure 1:**
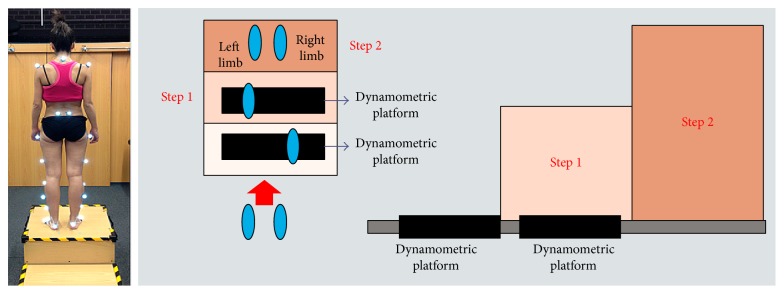
Portable two-step wooden staircase and two independent dynamometric platforms were used to perform the kinetic analysis during the stair ascent test.

**Figure 2:**
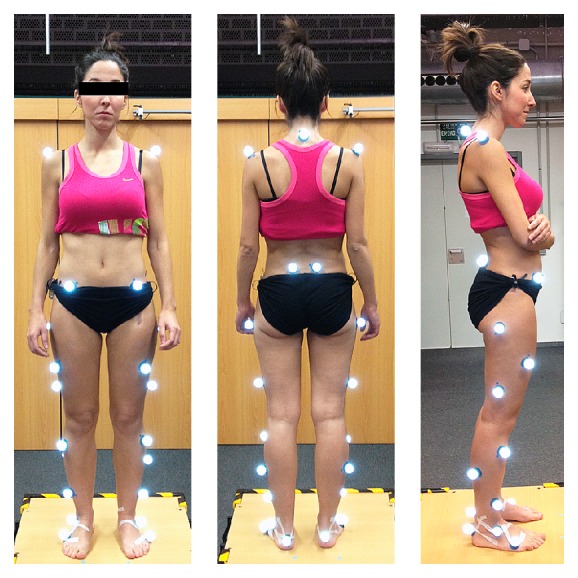
Subject with reflective markers used for kinematic analysis.

**Figure 3:**
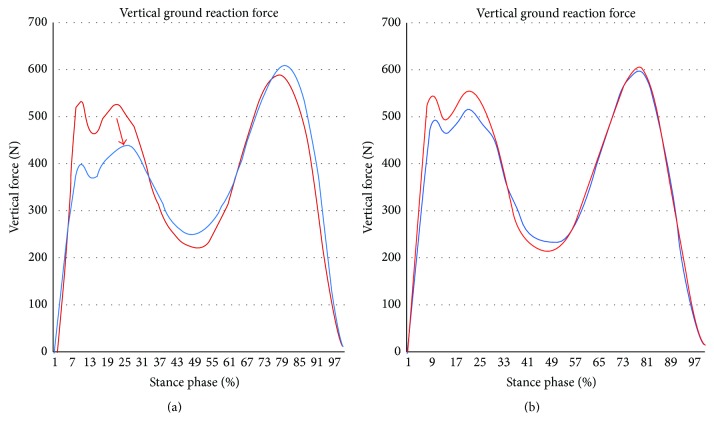
Gait analysis. (a) Preoperative. (b) Postoperative. Red line, right lower limb. Blue line, pathologic left lower limb. Preoperative study shows a decrease of the vertical heel contact force (arrow) that could be a defense mechanism to avoid the load in the pathologic limb. Notice the gait pattern normalization after surgery.

**Figure 4:**
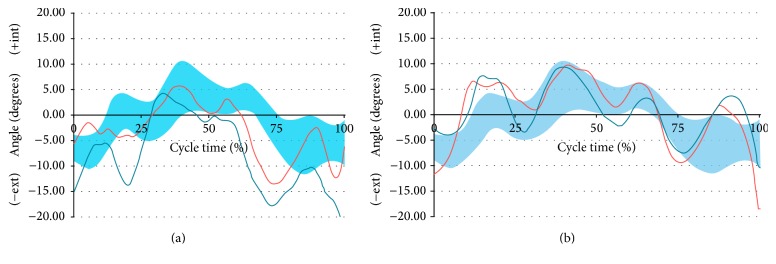
Kinematic gait analysis. (a) Preoperative. (b) Postoperative (7 months of follow-up). Red line, right hip. Blue line, pathologic left hip. Blue band, band of normality. Notice how the preoperative nonpathological hip values differ from those of the postoperative ones of the same hip. This is due to the fact that in the kinematic and kinetic studies the pathological limb influences the healthy limb. What is relevant is that after surgery, the values of both hips are in the normality band. Furthermore, the external rotation of the hip that has been operated on has decreased regarding the preoperative status.

**Figure 5:**
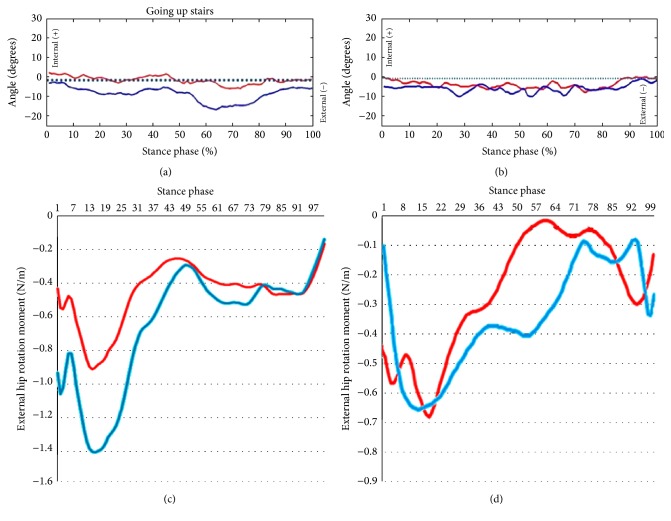
Kinematic analysis during stair ascending test. (a) Preoperative. (b) Postoperative (7 months of follow-up). Red line, right hip. Blue line, pathologic left hip. Kinetic analysis during stair ascending test. (c) Preoperative. (d) Postoperative (7 months of follow-up). Red line, right hip. Blue line, pathologic left hip. In the *x*-axis you can note the stance phase percentage. Stance phase begins with the heel strike and ends with the toe off.

For documentation purposes prior to hip surgery, she was evaluated using kinetic (gait analysis and hip rotation moment) and kinematic (rotating hip excursion) analyses during gait and stair ascent as the latter activity was the one that brought about a major limitation in her daily life. A pathway with two extensometric force plates on its surface was used to carry out the gait analysis. The subject was asked to walk at a high cadence rate because the faster the subject walks, the more evident the functional impairment becomes. The subject was required to walk at the same cadence before and after surgery. Before the data were collected, the subject walked on the pathway several times until she was able to walk with a natural and constant gait. A portable two-step wooden staircase and two independent dynamometric platforms, placed as indicated in Figure 1, were used to perform the kinetic analysis during the stair ascent test. An eight-camera computer-aided video motion analysis system and reflective passive markers that determined the spatial position of the segments of the lower limb were used to carry out the kinematic analysis (Figure 2). All of the markers were placed on the lateral aspect of the leg to allow for a correct visualization by the cameras (Figure 2). The kinetic and kinematic parameters were analyzed using the NedRodilla/IBV software (Instituto de Biomecánica de Valencia, Valencia, Spain). Preoperative gait analysis showed an altered gait pattern (Figure 3). Preoperative kinematic analysis showed a gait (Figure 4(a)) and stair ascent (Figure 5(a)) pattern with external rotation of the involved hip. Moreover, hip external rotation torque of the involved hip increased significantly during stair ascent (Figure 5(c)).

During arthroscopy we confirmed the impingement mechanism with the hip at 90° of flexion and maximum internal rotation. With external femoral rotation we avoid the impingement and, in theory, the hip pain. We performed a femoral neck osteoplasty and reattachment of the labrum. After hip surgery, no specific physiotherapy treatment for the AKP was performed. At 6 months after surgery, the patient had virtually no discomfort in the hip, and knee pain had completely disappeared.

At 7 months kinetic and kinematic analyses were performed to evaluate the effects of hip surgery on the preoperative biomechanical parameters. They showed a normal gait pattern (Figure 3(b)) and a symmetric pattern between both hips (Figures 4(b), 5(b), and 5(d)).

At final follow-up (26 months) the patient was completely asymptomatic; both hip and knee and activities that previously could not be done or had been done with much difficulty like walking at a high cadence rate, going up or down stairs, squatting, making turns with the hip or using a car with clutch were now done without any problem. Moreover, she had begun running without any limitation. A Dunn radiograph view showed an alpha angle of 32°. The postoperative Kujala Knee Score was 91 and the postoperative Nonarthritic Hip Score was 97.50. Postoperative pain intensity on Visual Analogue Scale was 1 for the knee and 0 for the hip.

## 3. Discussion

The most important finding of this study was the link between abnormal hip function, in our case Cam FAI, and AKP in a young active patient.

Consistent with our hypothesis, our preoperative kinematic analysis demonstrated increased hip external rotation during gait and stair ascent, when compared to pain-free contralateral hip. This might be explained by the ball-and-socket configuration of the hip joint that allows the femur a high degree of mobility. In theory, this excessive hip external rotation was functional given that it was associated with hip internal rotation weakness as we were able to deduce from our kinetic analysis. Moreover, hip external rotation torque increased significantly during stair ascent, which explains hip external rotation during daily life activity. These kinetics findings are also in concurrence with our hypothesis. Moreover, our findings are also in agreement with a growing body of literature linking abnormal femur rotation and AKP [9–15].

AKP has been more frequently described in patients with internal femoral rotation. Most studies analyze the importance of internal femoral rotation in the genesis of AKP, but there are few that focus on external femoral rotation as occurs in our case. Lee et al. [10, 11] have performed the most cogent study that demonstrated the importance of femoral rotation in the genesis of AKP. They have found that an external rotational deformity of the femur causes an increment of the patellofemoral contact pressure on the medial facet of the patella. Yildirim et al. [13] have observed that an external rotational deformity of the femur greater than 10° provokes a significant medial tilt of the patellofemoral joint. This abnormal loading of the patellofemoral joint may be an important factor in the genesis of AKP. These findings also suggest that the control of femur rotation may be important to restoring normal patellofemoral joint kinematics. Cibulka and Threlkeld-Watkins [14] reported an unusual case of patellofemoral pain in a patient with excessive hip external rotation. Karaman et al. [9] have shown that a femoral rotational malalignment greater than or equal to 10°, both external and internal, after closed intramedullary nailing of femoral shaft fractures, affected the patellofemoral joint so as to provoke AKP while climbing stairs. A possible mechanism for this pain might be the patellofemoral imbalance brought on by the torsional deformity of the femur. In this study, the patients also suffered hip pain.

As occurs in other joints, functional tests used to evaluate Cam FAI could be designed to reproduce the symptoms (e.g., the classical impingement test) or to provoke “avoidance behavior” to protect against pain, which likewise could be interpreted as a positive sign. Kinematic and kinetic analyses allow us to evaluate this “avoidance behavior” under realistic loading conditions. Souza et al. [12] have focused on the importance of weight-bearing in patellofemoral joint kinematics and femur rotation. In our case, the “avoidance behavior” was a gait with external rotation of the lower limb as a compensatory strategy to reduce impingement and therefore pain. Kinematic and kinetic analyses help us to improve our knowledge of the aetiopathogeny of AKP and therefore might allow us to carry out a suitable treatment for these patients. A clear understanding of the cause of patellofemoral mal-tracking is crucial to nonsurgical as well as surgical treatment. In our case, the near normalization of the hip kinematic and kinetic parameters after the treatment of the Cam FAI and their correlation with the clinical improvement of AKP supports our hypothesis. The primary cause of AKP was in the hip.

Emphasis must be placed on the fact that we are dealing with a single case; that is why the results only have relative value. However, our observation has led us to initiate a prospective study in a large cohort of patients with AKP to analyze the prevalence of Cam FAI in this population in order to draw definitive conclusions.
